# Molecular characterization of *Fasciola hepatica* in endemic regions of Colombia

**DOI:** 10.3389/fvets.2023.1171147

**Published:** 2023-06-09

**Authors:** Diego Garcia-Corredor, Mateo Alvarado, Martín Pulido-Medellín, Marina Muñoz, Lissa Cruz-Saavedra, Carolina Hernández, Julio Cesar Giraldo, Luis R. Vásquez-Arteaga, Ana Cruz Morillo Coronado, Juan David Ramírez

**Affiliations:** ^1^Grupo de Investigación en Medicina Veterinaria y Zootecnia (GIDIMEVETZ), Facultad de Ciencias Agropecuarias, Universidad Pedagógica y Tecnológica de Colombia (UPTC), Tunja, Colombia; ^2^Centro de Investigaciones en Microbiología y Biotecnología-UR (CIMBIUR), Facultad de Ciencias Naturales, Universidad del Rosario, Bogotá, Colombia; ^3^Centro de Tecnología en Salud (CETESA), Innovaseq SAS, Bogotá, Colombia; ^4^Grupo de Investigación en Parasitología y Microbiología Tropical, Programa de Biología, Universidad INCCA de Colombia, Bogotá, Colombia; ^5^Facultad de Medicina y Ciencias de la Salud, Universidad Militar Nueva Granada, Bogotá, Colombia; ^6^Centro de Estudios en Microbiología y Parasitología, Facultad de Ciencias de la Salud, Universidad del Cauca, Popayán, Colombia; ^7^Universidad Pedagógica y Tecnológica de Colombia, Facultad de Ciencias Agropecuarias, Tunja, Colombia; ^8^Molecular Microbiology Laboratory, Department of Pathology, Molecular and Cell-Based Medicine, Icahn School of Medicine at Mount Sinai, New York, NY, United States

**Keywords:** *Fasciola hepatica*, phylogeneitc tree, Colombia, genetic diversity, population structure

## Abstract

*Fasciola hepatica* is a zoonotic trematode that affects a wide range of hosts, including cattle, sheep, and goats. The economic impact of the parasite on the cattle industry is significant, with high losses reported worldwide. While its impact on human health was previously underestimated, recent years have seen a rise in fascioliasis cases, leading to increased interest among researchers globally. To characterize the genetic diversity and intraspecific variation of this parasite in South America, specifically in Colombia, we collected 105 adult parasites from cattle bile ducts in seven Colombian departments (Antioquia, Boyacá, Santander, Cauca, Cundinamarca, Nariño, Norte de Santander, and Santander) to assess the parasite’s phenotypic analyses, genetic diversity, and population structure. A computer image analysis system (CIAS) was applied based on standardized morphological measurements. Liver-fluke size was studied by principal component analysis (PCA). DNA sequences were obtained for nuclear markers such as the 28S, β-tubulin 3, ITS1, ITS2, and the mitochondrial marker Cytochrome Oxidase I (COI). Multiple statistical tests were performed, and the parasite’s population structure was analyzed. Maximum Likelihood (ML) phylogenetic reconstructions were carried out using the sequences obtained herein and sequences available in GenBank. Morphological results revealed that all the obtained individuals matched *F. hepatica*’s morphology. There was no evidence of high genetic diversity, and the absence of genetic structure at the country-level was notable, possibly caused by a demographic expansion of this trematode in Colombia or the low resolution of the molecular markers employed. Future studies are still needed to unveil the genetic population structure of *F. hepatica* across the country.

## Introduction

*Fasciola hepatica* (1) a hepatic trematode, is a pathogen that affects both cattle and humans, causing a parasitic disease called fascioliasis (2). Its high pathogenicity during the disease’s invasive or acute phase, and during bile or chronic phase in cattle, sheep, and goats, causes huge economic losses of approximately $200 million USD per year. This is due to the confiscation of livers in slaughterhouses, weight gain reduction, and milk production (3–7). Recent research highlights the importance of the disease in human health, with the World Health Organization including it in their roadmap of neglected tropical diseases for 2030, and promoting the use of One Health strategies as a transversal approach (8, 9).

The global distribution of fascioliasis is wide, resulting from both the historical movement of Old-World animals during colonization and the geographic distribution of Limneidae snails, which act as intermediate hosts for the parasite (10). As a result, the prevalence of the disease in cattle varies widely worldwide. In Africa, prevalence ranges from 1.2 to 91%, while in the Americas, it varies between 24.5 and 100%. In Asian countries, prevalence values fluctuate from 0.71 to 69.2%, while in Papua New Guinea and Australia, the values range from 26.5 to 81%. In Europe, the prevalence varies between 0.12 and 86% (11).

The distinctive biology of *Fasciola hepatica* can affect its genetic diversity and structure (12). Within these biological parasites clonal expansion occurs inside the intermediate host (13), hence there is a possibility of the coexistence of multiple metacercariae sharing origin and genotype, and consequently, parasites sharing multilocus genotypes between definitive hosts (14). Additionally, as a hermaphrodite, the parasite has the potential to induce changes in the allele frequency of a population, and clonal expansion could involve a founder effect, resulting in changes in population structure (15).

Considering the complexity of *Fasciola* characterization through morphological examination (16), molecular approaches have been recently used to identify this parasite with higher accuracy. A variety of molecular markers, such as mitochondrial cytochrome oxidase I (COX1) and NADH dehydrogenase subunit 1 (NAD1), nuclear (28S rRNA) genes, and ribosomal internal transcribed spacers (ITS1 and ITS2), have proven useful for detecting hybrid forms of *Fasciola* (17). While molecular strategies have facilitated the identification of morphologically similar parasites (10), it is not yet the standard, and the distribution of some parasitic species is still unknown.

*Fasciola hepatica* is a significant economic burden in Colombia, causing losses of around $479,962 USD (18). The parasite is endemic in four recognized regions: Nariño, Cundiboyacense highlands, Santander, Norte de Santander highlands, and highlands of the west of Antioquia (19). The prevalence of *F. hepatica* varies across these areas, with values ranging from 9.5 to 30.9% (20–25). However, knowledge of the parasite’s genetic diversity and intrapopulation structure in the country is limited. Thus, this study aims to genetically characterize *F. hepatica* infecting cattle and analyze its population structure in seven departments of Colombia (Antioquia, Boyacá, Santander, Cauca, Cundinamarca, Nariño, Norte de Santander, and Santander), located in endemic biogeographic regions of the parasite.

## Methods

### Sample collection

**Figure 1:**
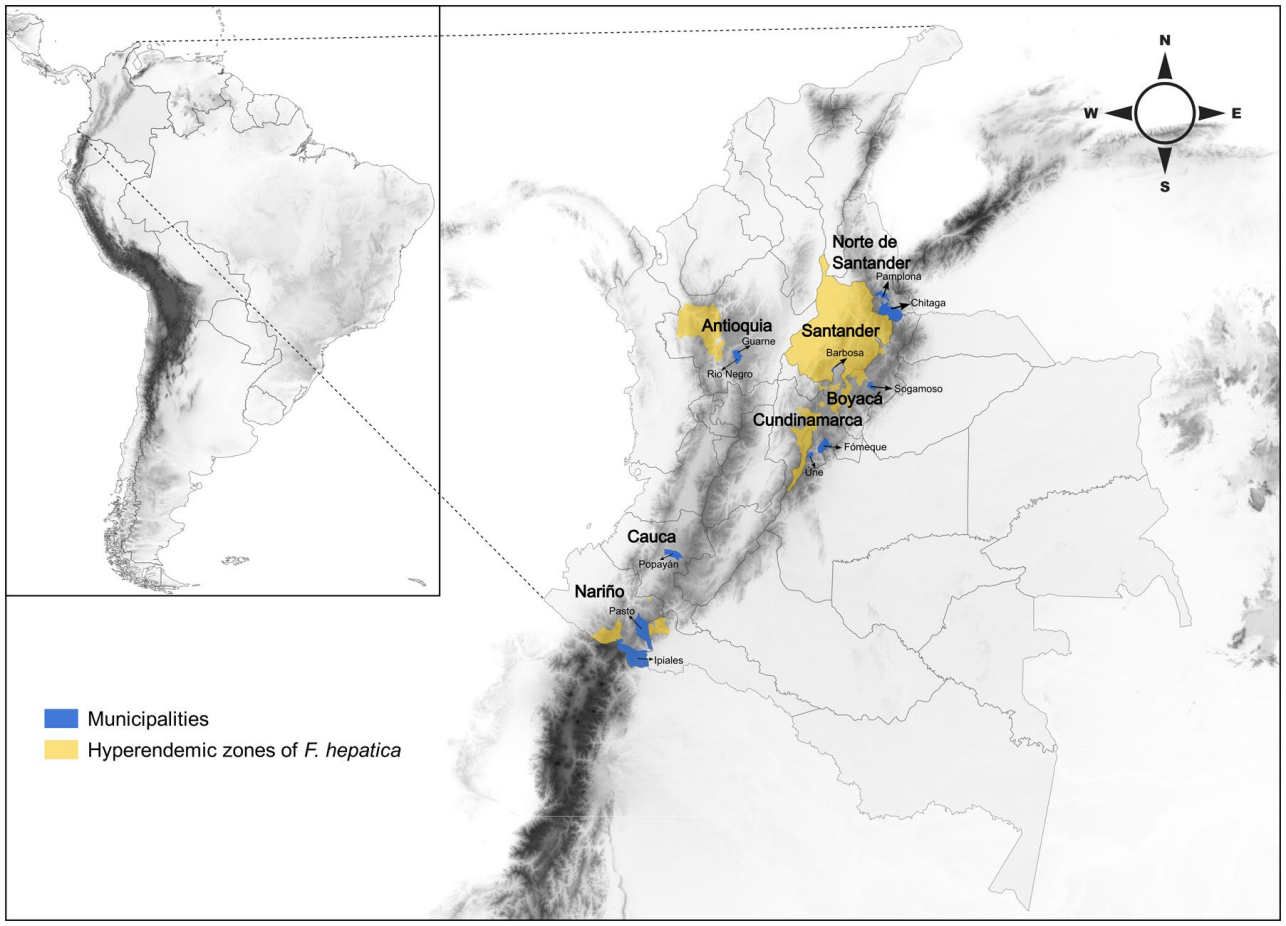
Map showing the municipalities where the collection of *Fasciola hepatica* adults was carried out, and the endemic zones for Colombia.

This study was conducted in seven departments of Colombia from 2021 to 2022: Antioquia, Boyacá, Santander, Cauca, Cundinamarca, Nariño, Norte de Santander, and Santander (Figure 1; Supplementary Table S1). During liver inspection of sacrificed animals, 15 *F. hepatica* adult samples were selected from the bile duct of 15 different cattle in each department (1 adult parasite per cattle). The flukes were washed with saline solution to remove bile residues and blood remains adhered to the parasite (26). Samples were preserved in 70% ethanol and refrigerated at 4°C to conduct the phenotypic analysis and then subjected to DNA extraction. Epidemiological cards were designed to allow data collection for each animal. This information was obtained from the Sanitary guides for the internal movement of animals (GSMI; Guías Sanitarias de Movilización Interna de Animales) issued by the Instituto Colombiano Agropecuario (Supplementary Table S2).

### Ethical statement

The current study was approved by the ethics committee of the Universidad Pedagógica y Tecnológica de Colombia with the title “Caracterización molecular y análisis de la estructura genética poblacional de *Fasciola* spp. en cinco departamentos de Colombia,” under report number 007/2019.

### Phenotypic analyses

Standardized measurements of *Fasciola* samples were made according to the methods proposed by Valero et al. (27) and Periago et al. (16, 28). The following lineal biometric characters were measured: body length (BL), maximum body width (BW), maximum diameter of oral sucker (OSmax), maximum diameter of ventral sucker (VSmax), distance between the anterior end of the body and the ventral sucker (A-*VS*), and distance between the ventral sucker and the posterior end of the body (*VS*-P). Additionally, areas were measured, including body area (BA), oral sucker area (OSA), and ventral sucker area (VSA), and the ratio of oral sucker area over ventral sucker area (OSA/VSA) was calculated.

Measurements were taken using a microscope and captured with a digital camera (Zeiss Primotech, Germany) and analyzed with image analysis software (Zeiss Zen 3.1 Blue Edition, Jena, Germany). Univariate morphometric comparisons were applied to calculate phenotypic variations among Fasciolid adults from each department and between departments to compare them with previous reports, excluding the effect of ontogenetic variations within the group (29). Reference values of Altiplano Bolivia, Cajamarca (Peru) and San Juan (Ecuador), Valencia (Spain), Córcega (France) and Bobo Dioulasso (Burkina Faso) for *F. gigantica* (16), are shown in Supplementary Table S3. Principal component analysis (PCA) was used to summarize the majority of the variations in a multivariate data set in a few dimensions (30). Results were considered highly significant when *p* < 0.01. Non-redundant measures (measures not included in another one) used were BL, BW, OSmax, VSmax, A-*VS*, and *VS*-P.

### Extraction, amplification, and alignment of DNA data

Genomic DNA was extracted from 105 adult *F. hepatica* parasites using the Invisorb® Spin Universal Kit (Statec Molecular) following the manufacturer’s protocol. The concentration of the extracted DNA was assessed using a NanoDrop ND-1000 spectrophotometer, while quality and integrity assessments were performed using electrophoresis with a 1% agarose gel. Minimum quality and integrity parameters were established to select the samples for further analysis, including DNA concentrations of at least 200 ng/μl and quality ratios between 260/280 of 1.7–2.

Molecular markers included in this study were amplified by PCR: *28S rRNA* (FAS-28sFwd-FAS-28sRV) (31), *β-tubulin 3* (FAS-BtubFwd-FAS-BtubRV) (32), Internal Transcribed Spacer 1-*ITS1* (FAS-ITS1Fwd-FAS-ITS1RV), Internal Transcribed Spacer 2-*ITS2* (FAS-ITS2Fwd-FAS-ITS2RV) (33), and Cytochrome Oxidase Subunit 1-*COI* (FAS-COIFwd-FAS-COIRV) (34). The sequences of the primers are shown in Supplementary Table S4, where fragments of 520, 836, 498, 364, and 438 bp were generated, respectively. Each PCR reaction consisted of a mixture of GoTaq Green Master Mix (Promega, Madison, WI, United States) at 1× concentration (400 μM dATP, 400 μM dGTP, 400 μM dCTP, 400 μM dTTP, and 3 mM MgCl2), 2.5 μM of each primer, 3 μL of total DNA, and 4.5 μL of molecular biology-grade water to complete a final volume of 25 μl.

PCR cycles were conducted with the following thermal profiles: (i) *28S*: starting denaturation at 94°C for 3 min, followed by 30 cycles of denaturation at 94°C for 30 s, then 30 cycles of annealing at 60°C for 30 s, 30 cycles of extension at 72°C for 60 s, and a final extension at 72°C for 5 min; (ii) *β-tubulin 3*: starting denaturation at 95°C for 2 min, followed by 35 cycles of denaturation at 95°C for 60 s, then 35 cycles of annealing at 55°C for 60 s, 35 cycles of extension at 72°C for 60 s, and a final extension at 72°C for 10 min; (iii) *ITS1*: starting denaturation at 94°C for 5 min, followed by 30 cycles of denaturation at 94°C for 30 s, then 30 cycles of annealing at 55°C for 30 s, 30 cycles of extension at 72°C for 2 min, and a final extension at 72°C for 10 min; (iv) *ITS2*: starting denaturation at 94°C for 2 min, followed by 35 cycles of denaturation at 93°C for 60 s, then 35 cycles of annealing at 55°C for 60 s, 35 cycles of extension at 72°C for 60 s, and a final extension at 72°C for 2 min; (v) *COI*: starting denaturation at 94°C for 90 s, followed by 30 cycles of denaturation at 94°C for 90 s, then 30 cycles of annealing at 55°C for 90 s, 30 cycles of extension at 72°C for 2 min, and a final extension at 72°C for 10 min. Amplicons were visualized using a 1.5% agarose gel.

Purification of PCR products was performed using ExoSAP-IT™ PCR Product Cleanup Reagent (Applied Biosystems, Foster City, CA, United States) following the manufacturer’s protocol, including quality and integrity DNA control. The purified products were then sequenced using Sanger sequencing. The resulting raw sequences were analyzed and contigs were assembled, verified, and edited in DNAStar Lasergene V7.1.0 (DNAStar, Inc., Madison, WI). The alignment of locus sequences, visual inspection, and manual correction of alignments were performed using Mesquite (35).

### Molecular phylogenetic and population genetics analyses

To characterize the genetic variability of *F. hepatica*, we estimated the genetic divergences and calculated the number of haplotypes (h), haplotype diversity (hd), nucleotide diversity (π) and number of segregating sites (S) using only the Colombian sequences (Supplementary Table S1) for each marker in DNASP v6.12.03 (36). We reconstructed phylogenetic relationships within the multiple *F. hepatica* samples only for markers that were informative according to the estimated genetic divergence calculations, using maximum likelihood (ML) inference on IQ-Tree 2 (37). The best substitution models for each locus were selected using ModelFinder (38), included in IQ-Tree 2, and considering the Bayesian Information Criterion for the final selection (BIC) (39). Therefore, the resulting substitution models for each locus were F81 + F for Cytochrome oxidase I (*COI*) and K2P + G4 for *β-tubulin 3*. We used UltraFast Bootstrap (40), aBayes (41), and SH-aLRT (42) to assess node support, performing each reconstruction with 1,000 pseudoreplicates. For these reconstructions, we included *Schistosoma turkestanicum* sequences obtained from GeneBank as the outgroup (Supplementary Table S5). TCS haplotype networks were constructed for both markers using PopArt v1.7 (43). As little to no intraspecific diversity was evident and no phylogeographic signal was detected in the first round of the reconstructions, we decided to include additional *F. hepatica* sequences from different countries retrieved from GenBank (Table 1) and re-run the reconstructions under the same parameters described above. The aim of the inclusion of new sequences was to determine if our sequences would cluster among themselves at a different geographic scale, indicating hypothetically that there is intraspecific diversity in *F. hepatica* at a larger geographic scale and not at the regional scale as we expected. We constructed a TCS haplotype network (47) for the *COI* and *β-tubulin 3* markers using PopArt v1.7 (43), including the new *F. hepatica* sequences from GenBank. Finally, we performed a principal coordinate analysis PCoA using the *COI* and *β-tubulin 3* alignments. To do this, we obtained a “dist” file that contained the Euclidean distances of these data sets. We then used the gl.pcoa function from the dartR package to conduct the analysis. To create the graphs, we utilized the colorplot function of the adegenet package.

**Table 1:** Accession numbers of *Fasciola hepatica* sequences used for phylogenetic and haplotype analyses.

Marker	N°	Accession number	Country	Reference
28S	1	MN970007	Australia	Le et al., Unpublished
	2	MF678654	Australia	Calvani et al. (17)
	3	HM369302	Bulgaria	Teofanova et al. (32)
	4	KF791538	Egypt	Mohammad-Gobbah et al., Unpublished
	5	HM369311	Poland	Teofanova et al. (32)
	6	HM369358	Poland	Teofanova et al. (32)
β tub	1	HM535803	Bulgaria	Teofanova et al. (32)
	2	HM535813	Greece	Teofanova et al. (32)
	3	HM535962	Greece	Teofanova et al. (32)
	4	HM535806	Greece	Teofanova et al. (32)
	5	HM535842	Poland	Teofanova et al. (32)
ITS1	1	MN559388	Algeria	Amor et al. (48)
	2	AJ243016	Bolivia	Bargues et al. (49)
	3	MF991101	Iran	Heydarian et al., Unpublished
	4	EF612469	Iran	Lotfy et al. (50)
	5	MG569976	Mexico	Valero et al. (44)
	6	KJ689334	Peru	Reyna and Sanabria (51)
	7	KJ689322	Peru	Reyna and Sanabria (51)
	8	KJ689321	Peru	Reyna and Sanabria (51)
	9	KJ689320	Peru	Reyna and Sanabria (51)
	10	GQ231547	Tunisia	Farjallah et al. (52)
	11	GQ231546	Tunisia	Farjallah et al. (52)
ITS2	1	MG569985	Bolivia	Valero et al. (44)
	2	MT423007	Egypt	Khalafalla (53)
	3	KT033698	Iran	Shahbakhsh et al. (54)
	4	MG569976	Mexico	Valero et al. (44)
	5	MG569983	Mexico	Valero et al. (44)
	6	KJ852770	Peru	Reyna and Sanabria (51)
	7	MG569981	Spain	Valero et al. (44)
	8	MG569986	Spain	Valero et al. (44)
COI	1	MK838687	Brazil	Schwantes et al. (34)
	2	MK838686	Brazil	Schwantes et al. (34)
	3	MW867317	Ecuador	Bargues et al. (45)
	4	MN527599	Iran	Khazan et al. (55)
	5	MK447982	Iran	Javanmard et al., Unpublished
	6	MK447972	Iran	Javanmard et al., Unpublished
	7	MF788106	Iran	Heydarian et al., Unpublished
	8	KR422386	Poland	Norbury et al., unpublished
	9	MW867326	Uruguay	Bargues et al. (46)
	10	GU112483	United States	Ai et al. (56)

## Results

### Morphometric analysis

Table 2 presents the morphometric values of *F. hepatica*, including extreme values, mean ± standard deviation by department, from Antioquia, Boyacá, Cauca, Cundinamarca, Nariño, Norte de Santander, and Santander. The data obtained from comparative morphometric analysis shows that there are no significant differences between *Fasciola* measurements from different departments (*p* > 0.01). Therefore, the samples do not exhibit any morphometric variation between departments (Table 2). The values of *F. hepatica* for the assessed departments, with measures of morphological traits considered useful to distinguish between *F. hepatica* and *F. gigantica*, demonstrate that none of the evaluated characteristics overlap with *F. gigantica.*

**Table 2:** Comparative morphometric data (extreme values, mean ± standard deviation) of *Fasciola hepatica* studied: Antioquia, Boyacá, Cauca, Cundinamarca, Nariño, Norte de Santander, and Santander (Colombia).

Adult measurements	Antioquia	Boyacá	Cauca	Cundinamarca	Nariño	Norte de Santander	Santander	*p*
Body area, BA	70.77–214.77	70.24–219.34	86.03–210.81	61.18–215.41	64.62–218.08	57.71–193.56	61.72–218.02	
	142.07 ± 52.48	154.88 ± 57.64	150 ± 41.32	127.77 ± 50.92	132.77 ± 52.32	119.27 ± 42.8	140.77 ± 52.42	0.480
Body length, BL	10.41–23.41	10.88–23.99	12.33–23.72	10.18–23.83	10.67–22.56	10.39–23.57	10.41–22.46	
	16.12 ± 4.14	18.64 ± 4.6	17.93 ± 4.25	16.39 ± 4.8	17.36 ± 4.03	16.82 ± 4.05	16.74 ± 3.91	0.684
Body width, BW	6.43–10.7	5.03–11	5–10.94	5.32–10.86	5.03–10.18	5.23–10.81	5.01–11	
	8.72 ± 1.44	7.46 ± 1.97	8.26 ± 2.18	8.26 ± 2.03	6.9 ± 1.5	7.79 ± 1.77	7.15 ± 1.86	0.085
BL/BW ratio	1.04–2.77	1.05–2.87	1.09–3	1.15–2.89	1.02–2.96	1–2.81	1.1–2.98	
	1.98 ± 0.6	1.94 ± 0.6	2.19 ± 0.69	2.13 ± 0.59	2.18 ± 0.49	1.68 ± 0.58	2.21 ± 0.59	0.168
Oral sucker area, OSA	0.24–0.5	0.2–0.49	0.2–0.49	0.2–0.45	0.2–0.5	0.2–0.49	0.24–0.48	
	0.4 ± 0.08	0.31 ± 0.09	0.33 ± 0.08	0.3 ± 0.07	0.33 ± 0.09	0.38 ± 0.08	0.34 ± 0.08	0.068
Maximum diameter of the oral sucker, OSmax	0.5–1	0.58–1	0.5–0.98	0.52–0.93	0.5–1	0.59–0.97	0.5–0.94	
	0.7 ± 0.16	0.78 ± 0.13	0.72 ± 0.17	0.77 ± 0.1	0.77 ± 0.15	0.79 ± 0.1	0.73 ± 0.16	0.577
Ventral sucker area, VSA	0.52–1.4	0.53–1.38	0.63–1.4	0.51–1.32	0.55–1.21	0.65–1.38	0.5–1.4	
	1 ± 0.29	0.93 ± 0.3	1.06 ± 0.24	0.87 ± 0.23	0.84 ± 0.24	1.01 ± 0.27	0.86 ± 0.3	0.204
Maximum diameter of the ventral sucker, VSmax	0.76–1.36	0.8–1.4	0.71–1.4	0.71–1.31	0.71–1.39	0.74–1.4	0.82–1.39	
	1.16 ± 0.16	1.08 ± 0.22	1.02 ± 0.25	1 ± 0.19	1.01 ± 0.21	1.05 ± 0.18	1.08 ± 0.19	0.366
OSA/VSA ratio	0.25–0.78	0.28–0.78	0.31–0.75	0.26–0.8	0.28–0.78	0.27–0.79	0.26–0.74	
	0.52 ± 0.16	0.6 ± 0.13	0.53 ± 0.14	0.55 ± 0.2	0.55 ± 0.15	0.48 ± 0.15	0.56 ± 0.14	0.572
Distance between the anterior end of the body and the ventral sucker, A-*VS*	1.21–2.76	1.16–2.74	1.15–2.78	1.25–2.71	1.14–2.78	1.11–2.8	1.12–2.57	
	2.01 ± 0.55	1.92 ± 0.47	2.08 ± 0.58	1.84 ± 0.45	1.73 ± 0.53	1.99 ± 0.51	1.96 ± 0.45	0.587
Distance between the ventral sucker and the posterior end of the body, *VS*-P	12.28–25.73	13.49–25.15	13.24–25.99	12.22–21.92	14.54–24.89	13.16–23.88	12.26–25.39	
	18.79 ± 5.03	19.82 ± 3.83	18.17 ± 4.58	16.78 ± 3.55	19.94 ± 3.48	18.86 ± 3.66	17.04 ± 4.44	0.240

Lineal biometric characters in mm, areas in mm^2^ and ratios without units.

**Figure 2:**
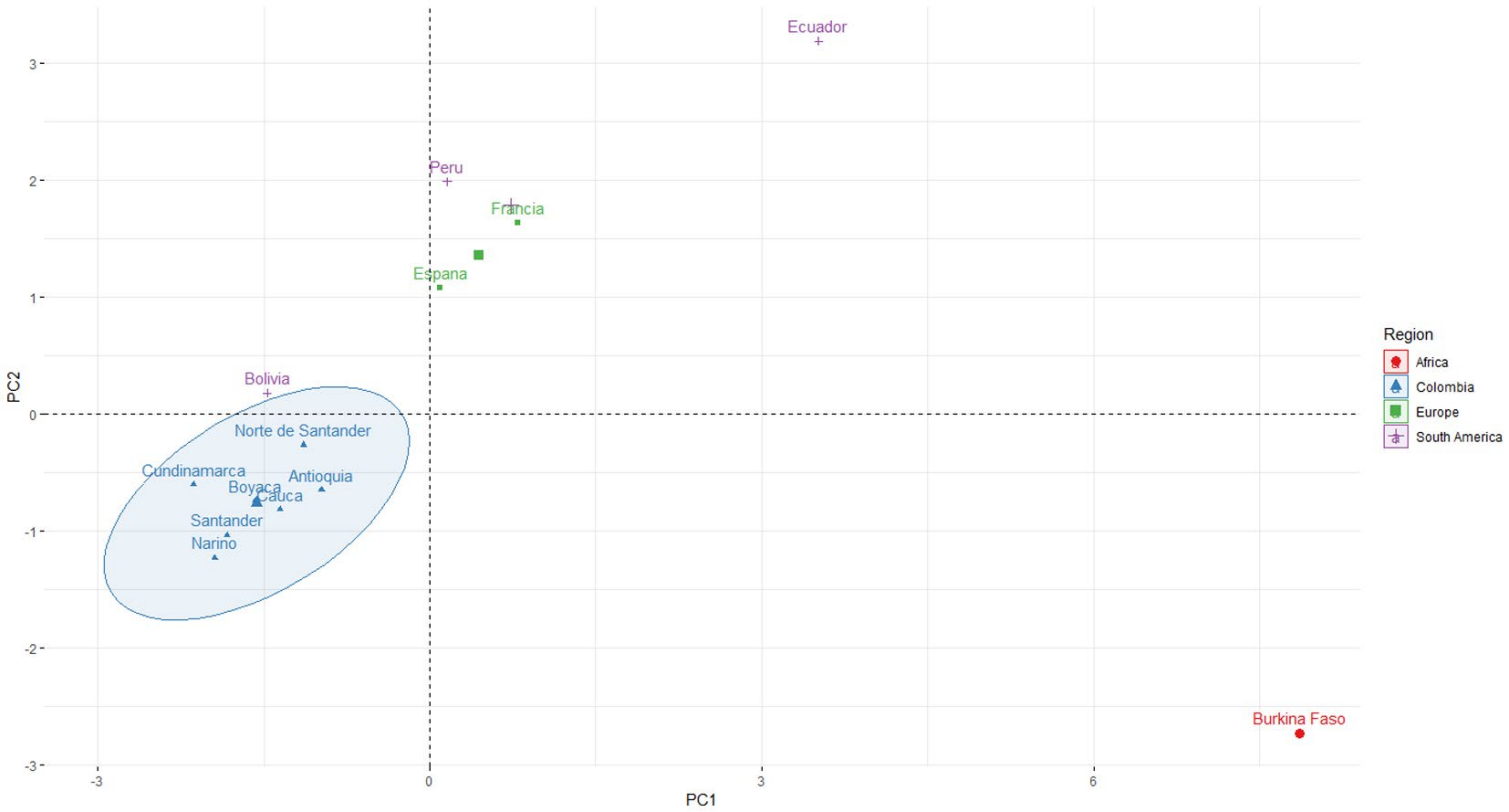
Plot for the comparison of *F. hepatica* specimens from Antioquia, Boyacá, Cauca, Cundinamarca, Nariño, Norte de Santander, and Santander (Colombia), with *F. hepatica* specimens from Altiplano Bolivia, Cajamarca (Peru), San Juan (Ecuador), Valencia (Spain), Corsica (France) and *F. gigantica* from Bobo Dioulasso (Burkina Faso). The samples are projected onto the first (PC1, 66.6%) and second (PC2, 21%) principal components.

In the dispersion graph of principal components (Supplementary Figure S1), the populations from the seven departments of this study were grouped in the same cluster, which was well separated from Burkina Faso’s *F. gigantica* but remarkably close to Bolivia’s *F. hepatica*. This suggests that the sizes of the populations from our study and those from the Bolivian highlands are similar. Additionally, the proximity between European and Peruvian populations was observed, while Ecuadorian populations appeared to be distant from the rest of the *F. hepatica* populations analyzed (Figure 2).

### Phylogenetic analysis

The analysis of genetic divergence showed that there was not a significant genetic variability in the *ITS1, ITS2*, and *28S* sequences of the Colombian samples. Although an attempt was made to concatenate the ribosomal markers, the genetic divergence calculations indicated an absence of genetic diversity. However, the *COI* and *β-tubulin 3* sequences showed a signal of genetic variability, with the *COI* sequences exhibiting less genetic variability compared to *β-tubulin 3* sequences (h 3; hd 0.648; π 0.00182 and h 10; hd 0.945; π 0.00843, respectively; Table 3).

**Table 3:** Population genetics summary statistics for each marker.

Marker	Statistic			
	h	hd	π	S
28S	2	0.143	0.00025	1
COI	3	0.648	0.00182	2
ITS1	1	0	0	0
ITS2	1	0	0	0
β tub 3	10	0.945	0.00843	25
28S + ITS1 + ITS2	1	0	0	0

h, number of haplotypes; hd, haplotype diversity; π, nucleotide diversity; S, number of segregating sites.

To assess genetic divergence, only *COI* and *β-tubulin 3* markers were found to be informative, leading to the decision to perform phylogenetic analyses solely for these markers. A preliminary analysis of haplotype networks and phylogenetic trees were conducted on Colombian samples, but due to the low genetic variability of *Fasciola* in Colombia, it was not possible to detect any genetic structure among the analyzed departments (Supplementary Figures S2, S3). The resulting topologies were not consistent, and there was no grouping between departments. An external sequence analysis was subsequently performed to determine if the Colombian sequences would cluster with themselves against others on a different geographical scale. The resulting topologies for both *COI* and *β-tubulin 3* show that the Colombian sequences form paraphyletic clades, intermingled with external sequences from GenBank included in the analysis, suggesting low genetic diversity in *Fasciola* at a continental scale and corroborating the results of the genetic diversity calculations. In both reconstructions, the small distance between the terminal branches and their corresponding nodes and the presence of single clades composed of identical sequences, likely separated from the rest of the sequences by one or two SNPs, indicate this low genetic diversity.

**Figure 3:**
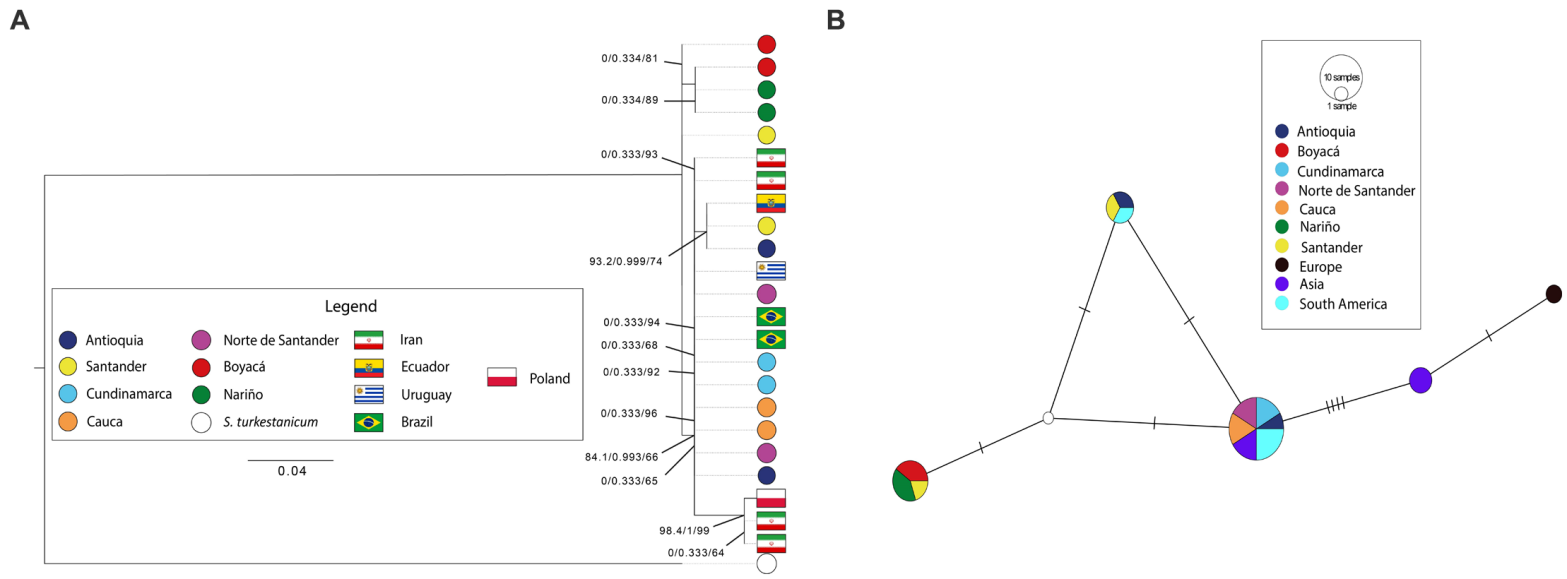
**(A)** Phylogenetic reconstruction with the ML algorithm based on the molecular marker *COI*. Flags represent external sequences included in this analysis. Bootstrap values on the internal nodes are shown in the following order: SH-aLRT/aBayes/UFBootstrap support. Only nodes with bootstrap values higher than 60 are shown. Black lines indicate a node’s bootstrap values. **(B)** *COI* TCS haplotype network. European haplotypes includes Poland’s sequence, Asian haplotypes includes Iran’s sequences and South American haplotypes include sequences from Brazil, Uruguay and Ecuador. Intermediate haplotypes shown as white circles.

**Figure 4:**
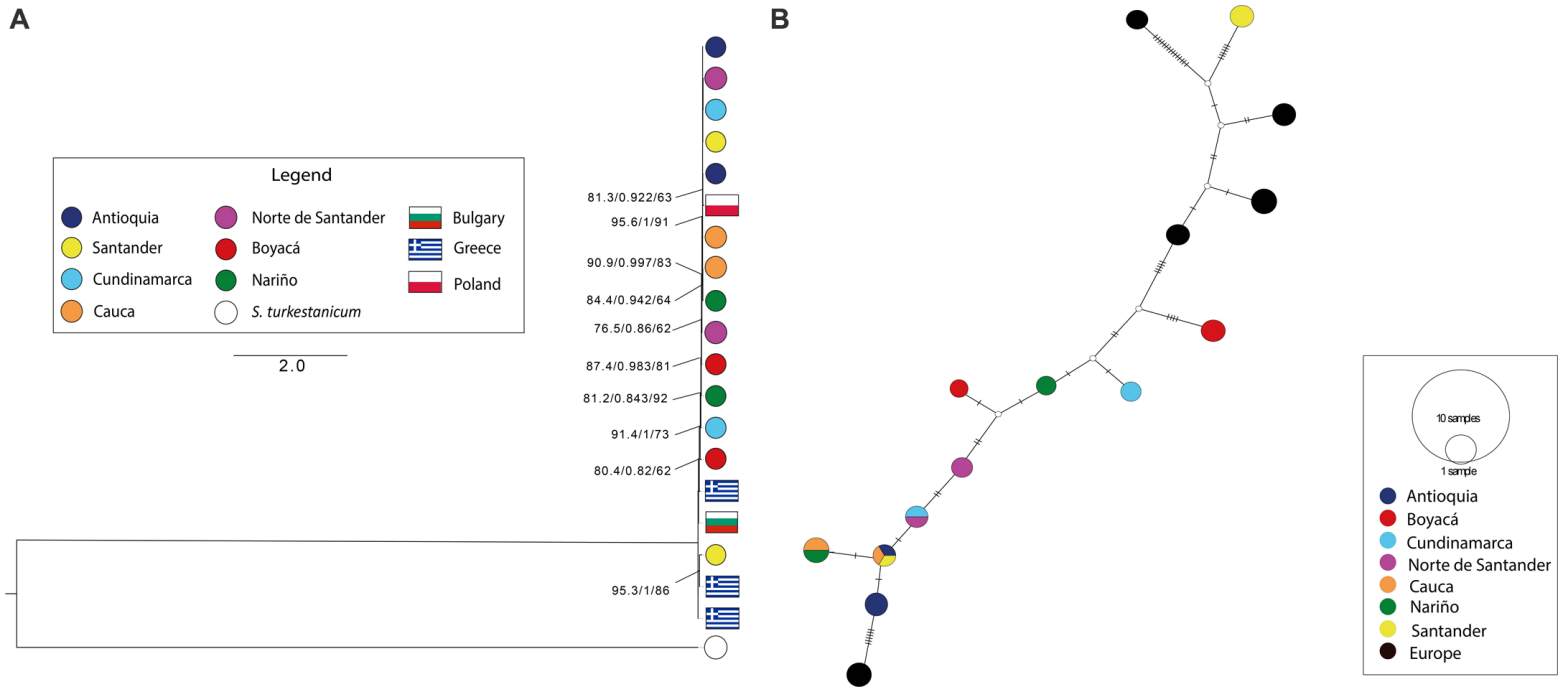
**(A)** Phylogenetic reconstruction with the ML algorithm based on the molecular marker *β-tubulin 3*. Flags represent the origin of external sequences included in this analysis. Bootstrap values on the internal nodes are shown in the following order: SH-aLRT/aBayes/UFBootstrap support. Only nodes with bootstrap values higher than 60 are shown. Black lines indicate a node’s bootstrap values. **(B)** *β-tubulin 3* TCS haplotype network. European haplotypes include sequences from Bulgary, Greece and Poland. Intermediate haplotypes are shown as white circles.

The *COI* haplotype network revealed a new haplotype in Boyaca, Nariño, and Santander, while two haplotypes previously reported in South America, and one of them also found in Asia. On the other hand, the *β-tubulin 3* haplotype network showed that new haplotypes were found in Cauca and Nariño; Antioquia, Cauca, and Santander; Cundinamarca, and Norte de Santander. Both haplotype networks, along with the phylogenetic reconstructions and genetic diversity calculations, demonstrated a low genetic diversity between the samples, with only a few mutational steps separating the different haplotypes detected. Furthermore, external sequences grouped with Colombian sequences in both haplotype networks, corroborating the results of the topologies obtained (Figures 3, 4). However, intraspecific diversity was higher in *β-tubulin 3* sequences than *COI* sequences (Supplementary Table S6). This was evident in the phylogenetic reconstructions and haplotype networks, where multiple clades and haplotypes were composed of a single sequence separated by a small distance or a small number of mutational steps. The *COI* dataset showed genetic structuring, which was not confirmed by the *β-tubulin 3* dataset, possibly due to differences in the genetic variability detected for the two markers.

The principal coordinates analysis graph shows that the sequences from the seven Colombian departments analyzed in this study were grouped together in the same clusters, for both *COI* and *β-tubulin 3* markers, and were clearly distinct from sequences from Asia, Europe, and South America (refer to Supplementary Figures S4, S5). This finding is consistent with the results of the previously described morphometric analyses. Together, the data from *COI* and *β-tubulin* 3 markers confirm the low genetic diversity observed in the morphological analyses at both the country and continental levels, and provide new insights into the low molecular diversity of Colombian *F. hepatica* samples. These results suggest that *F. hepatica* has low genetic diversity at the global scale.

## Discussion

This study represents the first comprehensive analysis of both the phenotype and genotype of *F. hepatica* in Colombia. Our morphological analysis of adult parasites revealed values consistent with those previously reported for *F. hepatica* in other regions of Europe and the Americas (Supplementary Table S3) (16, 44, 45), indicating the absence of *F. gigantica*. Despite being collected from different geographical areas characterized by highland environments with permanent transmission patterns, we did not observe any significant phenotypic differences between the parasites analyzed in this study. However, previous research has shown that intraspecific variability in *Fasciola* spp. can be linked to changes in altitude, as reported in studies from different regions (3, 8, 10, 57).

Valero et al. (44) found that in regions with high altitude, reduced oxygen levels induce hypoxia in hosts, affecting egg production, uterus development, and the size of the trematode body. As a result, egg production, uterus development, and the overall size of the parasite are significantly reduced in high-altitude regions, such as the Bolivian highlands where *F. hepatica* size is smaller than in Europe and other American regions (16, 46). Our study’s parasitic worms showed similar sizes to those reported in the Bolivian highlands (Table 2; Figure 2; Supplementary Table S3), indicating that the samples were collected from mountain ranges at altitudes between 2,050 and 2,569 meters above sea level (masl). Bargues et al. (45) mention that there is no apparent relationship between adult trematode shape and altitude or geographic location, but phenotypical changes are linked to the definitive host, with low persistence of morphological characteristics in subsequent infections.

Bargues et al. (45) suggested that there is no significant relationship between the shape of adult *F. hepatica* and altitude or location. However, our assessments of natural populations of *F. hepatica* allowed us to distinguish two phenotypic patterns: the valley pattern and the highlands pattern. Our findings indicate that populations of Andean valleys and European populations display phenotypic homogeneity, unlike highlands populations, which exhibit a wide size range with low values. This suggests that smaller sizes are sufficient to achieve gravidity in the uterus (58), resulting in reduced egg production compared to populations described in Mexico, Ecuador, and Europe (44, 45, 59, 60). Our study collected *F. hepatica* from highland zones, and our results align with Valero et al. (44) proposal, which observed smaller *F. hepatica* sizes in Antioquia, Boyacá, Cauca, Cundinamarca, Nariño, Norte de Santander, and Santander in relation to European samples. This is consistent with the transmission patterns and epidemiology of fascioliasis in various geographical regions. For instance, in the northern highlands of Bolivia, the transmission of the disease is permanent due to stable temperatures throughout the year and the constant presence of water puddles (61). In this context, the permanent elimination of eggs becomes a priority to facilitate transmission throughout the year, as in the zones where our study was conducted. In contrast, in some Mexican regions, transmission of the trematode is seasonal (62), as in low altitude regions in Europe, where a larger uterus can store eggs during unfavorable seasons (60).

In our study, we found that the *28S, ITS1*, and *ITS2* markers were not informative when evaluating the levels of genetic variation. This is likely due to the high percentage of repeat sequences (63, 64), in the *Fasciola* genome, which leads to low-quality assemblies and difficulties in designing molecular markers that can provide better characterization of the parasite. While these markers are still being used, reports of low resolution are common in other countries (29, 45, 59, 65). To overcome this limitation, it is necessary to obtain a reference genome for *F. hepatica* that can be used to design more informative markers to reveal the parasite’s evolutionary history. Mitochondrial genes have been shown to be informative for phylogenetic studies of *F. hepatica* due to their high mutation rate (66, 67). However, in our study, the use of the *COI* marker did not allow us to reconstruct phylogenetic relationships within Colombian samples (Figure 3), a situation similar to that found by Chaouadi et al. (68) in samples obtained in Algeria. To achieve higher resolution, it may be useful to integrate other mitochondrial markers such as nad1, as suggested by Bargues et al. (5). Although the β-tubulin 3 marker did not allow us to reconstruct phylogenetic relationships among Colombian samples (Figure 4), this marker presents opportunities for new investigations related to pharmacological resistance processes. Previous studies have shown that β-tubulin is associated with resistance to triclabendazole in *F. hepatica* (69). Therefore, the diversity observed in this marker for the Colombian samples could be explored to analyze and understand the mechanisms of resistance to triclabendazole, which is an important factor to consider in the control of this parasite (70).

Previous studies have shown that there is genetic variability in *F. hepatica* specimens in Latin America, which are similar to those found in Europe (32, 44, 46, 71, 72). This is likely due to successive introductions of cattle from abroad during two historical periods. The first period was the colonial era, where European and Central American animals were brought and subsequently introduced towards South America through the Pacific coast or the terrestrial route from what is now Colombia and Venezuela to the rest of the South American countries. The latter route has been considered the most significant route in terms of the introduction probability of *F. hepatica* haplotypes into the continent. The second period was the post-colonial era, characterized by an increase in Imports of cattle from Europe, North America, and Asia to improve existing breeds in South American countries (45, 65). These introduction processes could have resulted in a wide haplotype diversity since metacercariae can infect different cattle species (73, 74). Additionally, *F. hepatica* infection does not generate premunition, leading to reinfections and the accumulation of the parasite inside the same host (75). This indicates that animal movements across borders could be the indirect source of introducing more than one haplotype capable of infecting multiple susceptible species. Therefore, using molecular tools as a diagnostic strategy in epidemiological surveillance protocols in border corridors is essential for *F. hepatica* identification.

During the colonial period in Colombia, cattle were distributed in both the plains and highlands of the country, similar to other Latin American countries. However, unlike other countries, there was a reduction in the number of cattle raisers during the independence period, and the remaining populations clustered around human dwellings. In the late 19th century, there was a significant increase in the number of cattle in Colombia, but with little participation from imported individuals. This suggests that the restoration of the cattle population in Colombia started from previously established individuals (76, 77). Despite the increased importation of stallions in the 20th century (78), the process of restocking and distribution of cattle in Colombia may have resulted in a founder effect that could explain the low genetic diversity of parasites, including *F. hepatica*, in the studied zones (15, 65). However, an archaeological study found evidence of *F. hepatica* in South America at least 2,300 years ago (79), which opens up a new hypothesis to be explored through molecular analysis of archaeological samples from other continents to clarify the time and route of entry of the parasite into South America. Nevertheless, further studies are still needed to explain the low genetic diversity of *F. hepatica* in Colombia.

The current study has provided new insights into the phylogenetic relationships and structure of *F. hepatica* in Colombia, revealing a low diversity of haplotypes for two markers. Despite the parasite’s reported presence in multiple regions of the country, the expected excess of haplotypes that typically accompanies geographic expansion is not observed (80), as Table 3 illustrates. These findings differ from those in other countries where *Fasciola* population expansion is evident, such as Ecuador, Argentina, and Uruguay. This discrepancy may be due to differences in the molecular markers used in characterization, as well as to factors such as the arrival and movement of cattle in each country, sociocultural aspects, cattle handling practices, and the presence and distribution of intermediate host species (46, 67, 81). Further studies are required to gather more information and confirm the hypothesis that the population structure of *F. hepatica* is influenced by the mobility of the parasite’s definitive host. In Colombia, the high mobility of cattle and other definitive hosts may result in a low population structure of *F. hepatica*, leading to a greater spread of the parasite. Insights gained from these studies will improve our understanding of the host-vector-pathogen triad and facilitate the management of fascioliasis by providing insights into the dynamics of the pathogen’s population structure.

This study is the first to characterize the genetic structure of *Fasciola* in Colombia. We analyzed multiple departments and found that *F. hepatica* is exclusively circulating in the country, without strong indications of genetic structure. However, to broaden our comparisons, more sampling efforts are required to include other regions, using our results as a reference. Furthermore, additional studies are necessary to obtain a reference genome and identify suitable molecular markers that can enhance our understanding of the evolutionary history of *F. hepatica* and complement our current findings. Research on Limneidae snails, which are essential in the parasite’s life cycle, is also necessary to better understand their distribution, implications, and potential role in the circulation of new haplotypes.

## Data availability statement

The datasets presented in this study can be found in online repositories. The names of the repository/repositories and accession number(s) can be found below: https://www.ncbi.nlm.nih.gov/genbank/, OQ518355-OQ518368, OQ513221-OQ513234, OQ513976-OQ513989, OQ532997-OQ533010, OQ513939-OQ513952.

## Author contributions

JR, DG-C, MP-M, and AC: conceptualization. MM, CH, MA, LC-S, DG-C, and JR: data curation. MA, DG-C, MM, and JR: formal analysis. DG-C, MP-M, JR, AC, and MM: funding acquisition. JR, DG-C, MM, MP-M, JG, LV-A, and MA: methodology. DG-C, MP-M, and JR: project administration. JR, DG-C, and MP-M: resources. CH, MA, DG-C, MM, and JR: software. MM and JR: supervision. JR, MM, and AC: validation. DG-C, MA, and JR: writing—original draft. MP-M, MM, LC-S, CH, MA, JG, LV-A, AC, and JR: writing—review and editing. All authors contributed to the article and approved the submitted version.

## Funding

This study was funded by the Vicerrectoría de Investigación y Extensión at Universidad Pedagógica y Tecnológica de Colombia, under grant SGI2828. The authors also extend their gratitude to the Dirección de Investigación e Innovación at Universidad del Rosario for their support.

## Conflict of interest

The authors declare that the research was conducted in the absence of any commercial or financial relationships that could be construed as a potential conflict of interest.

## Publisher’s note

All claims expressed in this article are solely those of the authors and do not necessarily represent those of their affiliated organizations, or those of the publisher, the editors and the reviewers. Any product that may be evaluated in this article, or claim that may be made by its manufacturer, is not guaranteed or endorsed by the publisher.
