# Supplementary material for: Molecular characterization of Fasciola hepatica in endemic regions of Colombia
Source: Front Vet Sci. 2023 Jun 9;10:1171147. doi: 10.3389/fvets.2023.1171147 (PMC10288157; doi:10.3389/fvets.2023.1171147)
Supplement: Supplementary file 1 [file Table_1.DOCX]

***S1 Table.*** *Characteristics of Colombian F. hepatica sequences used for phylogenetic and haplotype analyses.*

| **Marker** | **N°** | **Acc. N°** | **Sequence ID** | **Department** |
| --- | --- | --- | --- | --- |
| **28S** | 1 | OQ518355 | A10_28S_CS | Antioquia |
|  | 2 | OQ518356 | A11_28S_CS | Antioquia |
|  | 3 | OQ518357 | B5_28S_CS | Boyacá |
|  | 4 | OQ518358 | B15_28S_CS | Boyacá |
|  | 5 | OQ518359 | C2_28S_CS | Cundinamarca |
|  | 6 | OQ518360 | C11_28S_CS | Cundinamarca |
|  | 7 | OQ518361 | H7_28S_CS | Norte de Santander |
|  | 8 | OQ518362 | H15_28S_CS | Norte de Santander |
|  | 9 | OQ518363 | K1_28S_CS | Cauca |
|  | 10 | OQ518364 | K2_28S_CS | Cauca |
|  | 11 | OQ518365 | N10_28S_CS | Nariño |
|  | 12 | OQ518366 | N14_28S_CS | Nariño |
|  | 13 | OQ518367 | S8_28S_CS | Santander |
|  | 14 | OQ518368 | S15_28S_CS | Santander |
| **β-tubulin 3** | 1 | OQ513221 | A4_TUB_CS | Antioquia |
|  | 2 | OQ513222 | A10_TUB_CS | Antioquia |
|  | 3 | OQ513223 | B3_TUB_CS | Boyacá |
|  | 4 | OQ513224 | B8_TUB_CS | Boyacá |
|  | 5 | OQ513225 | C2_TUB_CS | Cundinamarca |
|  | 6 | OQ513226 | C10_TUB_CS | Cundinamarca |
|  | 7 | OQ513227 | H1_TUB_CS | Norte de Santander |
|  | 8 | OQ513228 | H12_TUB_CS | Norte de Santander |
|  | 9 | OQ513229 | K3_TUB_CS | Cauca |
|  | 10 | OQ513230 | K16_TUB_CS | Cauca |
|  | 11 | OQ513231 | N8_TUB_CS | Nariño |
|  | 12 | OQ513232 | N13_TUB_CS | Nariño |
|  | 13 | OQ513233 | S4_TUB_CS | Santander |
|  | 14 | OQ513234 | S14_TUB_CS | Santander |
| **ITS-1** | 1 | OQ532997 | A4_ITS1_CS | Antioquia |
|  | 2 | OQ532998 | A7_ITS1_CS | Antioquia |
|  | 3 | OQ532999 | B1_ITS1_CS | Boyacá |
|  | 4 | OQ533000 | B15_ITS1_CS | Boyacá |
|  | 5 | OQ533001 | C1_ITS1_CS | Cundinamarca |
|  | 6 | OQ533002 | C13_ITS1_CS | Cundinamarca |
|  | 7 | OQ533003 | H2_ITS1_CS | Norte de Santander |
|  | 8 | OQ533004 | H13_ITS1_CS | Norte de Santander |
|  | 9 | OQ533005 | K5_ITS1_CS | Cauca |
|  | 10 | OQ533006 | K16_ITS1_CS | Cauca |
|  | 11 | OQ533007 | N7_ITS1_CS | Nariño |
|  | 12 | OQ533008 | N12_ITS1_CS | Nariño |
|  | 13 | OQ533009 | S4_ITS1_CS | Santander |
|  | 14 | OQ533010 | S8_ITS1_CS | Santander |
| **ITS-2** | 1 | OQ513939 | A2_ITS2_CS | Antioquia |
|  | 2 | OQ513940 | A9_ITS2_CS | Antioquia |
|  | 3 | OQ513941 | B4_ITS2_CS | Boyacá |
|  | 4 | OQ513942 | B12_ITS2_CS | Boyacá |
|  | 5 | OQ513943 | C2_ITS2_CS | Cundinamarca |
|  | 6 | OQ513944 | C12_ITS2_CS | Cundinamarca |
|  | 7 | OQ513945 | H1_ITS2_CS | Norte de Santander |
|  | 8 | OQ513946 | H12_ITS2__CS | Norte de Santander |
|  | 9 | OQ513947 | K2_ITS2_CS | Cauca |
|  | 10 | OQ513948 | K15_ITS2_CS | Cauca |
|  | 11 | OQ513949 | N3_ITS2_CS | Nariño |
|  | 12 | OQ513950 | N7_ITS2_CS | Nariño |
|  | 13 | OQ513951 | S1_ITS2_CS | Santander |
|  | 14 | OQ513952 | S11_ITS2_CS | Santander |
| **COI** | 1 | OQ513976 | A6_COI_CS | Antioquia |
|  | 2 | OQ513977 | A9_COI_CS | Antioquia |
|  | 3 | OQ513978 | B4_COI_CS | Boyacá |
|  | 4 | OQ513979 | B9_COI_CS | Boyacá |
|  | 5 | OQ513980 | C1_COI_CS | Cundinamarca |
|  | 6 | OQ513981 | C6_COI_CS | Cundinamarca |
|  | 7 | OQ513982 | H2_COI_CS | Norte de Santander |
|  | 8 | OQ513983 | H6_COI_CS | Norte de Santander |
|  | 9 | OQ513984 | K11_COI_CS | Cauca |
|  | 10 | OQ513985 | K16_COI_CS | Cauca |
|  | 11 | OQ513986 | N9_COI_CS | Nariño |
|  | 12 | OQ513987 | N14_COI_CS | Nariño |
|  | 13 | OQ513988 | S1_COI_CS | Santander |
|  | 14 | OQ513989 | S4_COI_CS | Santander |
