# Supplementary material for: Molecular characterization of Fasciola hepatica in endemic regions of Colombia
Source: Front Vet Sci. 2023 Jun 9;10:1171147. doi: 10.3389/fvets.2023.1171147 (PMC10288157; doi:10.3389/fvets.2023.1171147)
Supplement: Supplementary file 2 [file Table_2.DOCX]

***S2 Table.*** *GSMI: Guía Sanitaria de Movilización Interna de Animales issued by the Instituto Colombiano Agropecuario.*

*
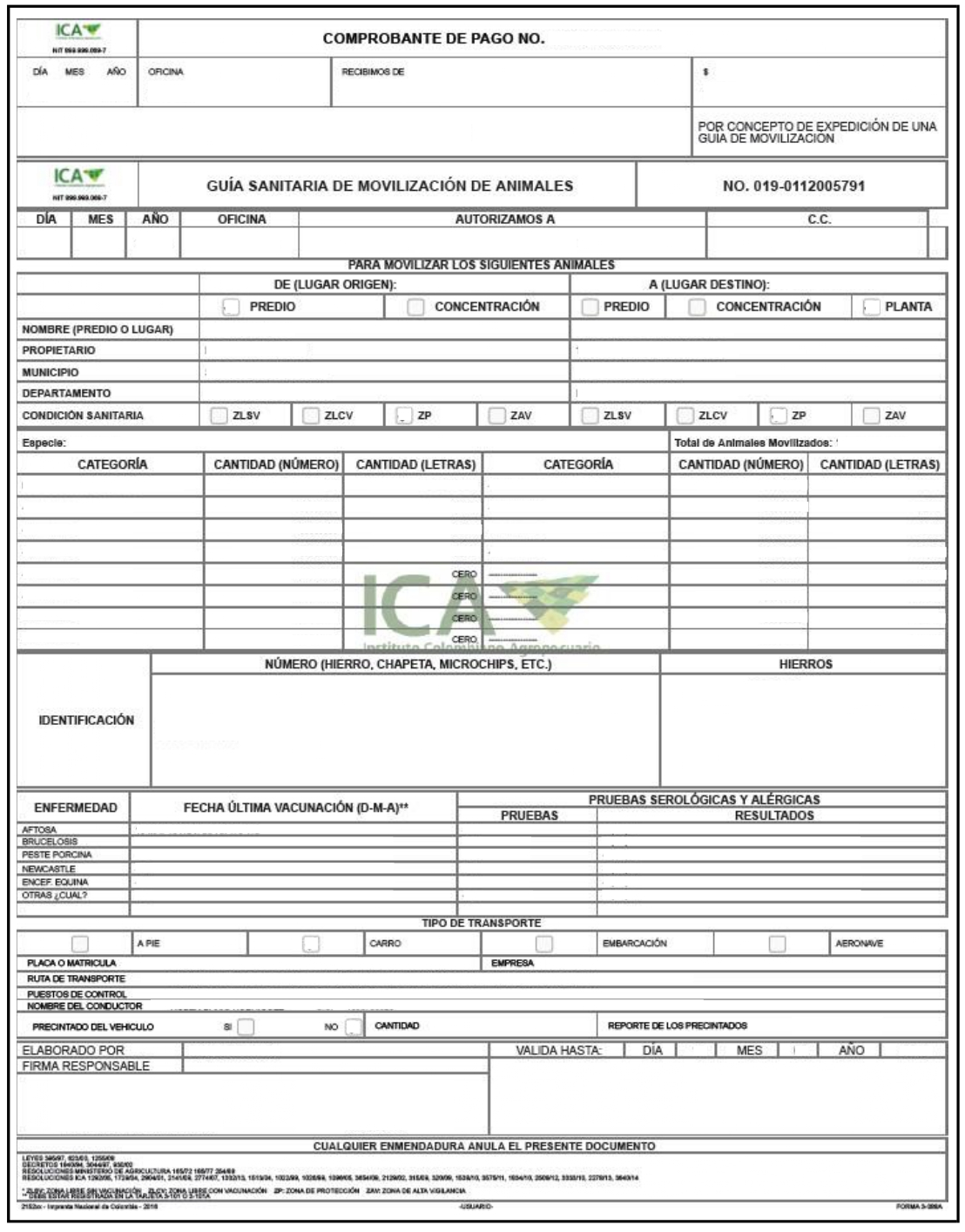
*
