# Supplementary material for: Molecular characterization of Fasciola hepatica in endemic regions of Colombia
Source: Front Vet Sci. 2023 Jun 9;10:1171147. doi: 10.3389/fvets.2023.1171147 (PMC10288157; doi:10.3389/fvets.2023.1171147)
Supplement: Supplementary file 3 [file Table_3.DOCX]

***S3 Table.*** *Comparative morphometric data (extreme values, mean ± standard deviation) of F.* *hepatica studied: Altiplano Bolivia, Cajamarca (Peru) and San Juan (Ecuador)^a^; Valencia (Spain) and Corsica (France)^b^; and F. gigantica studied in Bobo Dioulasso (Burkina Faso)^b^.*

| **Adult measurements** | **Bolivia** | **Peru** | **Ecuador** | **Spain** | **France** | **Burkina Faso** |
| --- | --- | --- | --- | --- | --- | --- |
| **Body area, BA** | 31.11 - 236.14 | 47.34 - 283.95 | 185,21 - 352.28 | 54.90 - 197.40 | 56.18 - 280.50 | 162.58 - 482.91 |
|  | 106.39 ± 3.35 | 135.87 ± 3.7 | 266.88 ± 4.7 | 126.85 ± 29.04 | 144.05 ± 55.72 | 257.01 ± 66.88 |
| **Body length, BL** | 9.64 - 31.04 | 13.48 - 30.97 | 21.49 - 32.78 | 11.64 - 22.93 | 15.43 - 29.00 | 28.81 - 52.29 |
|  | 18.08 ± 031 | 18.86 ± 0.31 | 28.38 ± 0.41 | 17.54 ± 2.08 | 20.39 ± 3.42 | 40.04 ± 5.16 |
| **Body width, BW** | 4.23 - 13.41 | 5.06 - 14.23 | 11.74 - 15.25 | 6.40 - 13.88 | 7.42 - 14.66 | 6.03 - 11.84 |
|  | 8.26 ± 0.14 | 10.25 ± 0.14 | 13.31 ± 0.14 | 10.10 ± 1.52 | 10.20 ± 1.81 | 8.54 ± 1.28 |
| **BL/BW ratio** | 1.41 - 3.74 | 1.31 - 3.73 | 1.68 - 2.62 | 1.29 - 2.77 | 1.48 - 2.53 | 3.39 - 6.77 |
|  | 2.22 ± 0.03 | 1.86 ± 0.03 | 2.14 ± 0.04 | 1.76 ± 0.26 | 2.01 ± 0.20 | 4.75 ± 0.72 |
| **Oral sucker area, OSA** | 0.21 - 0.66 | 0.20 - 0.67 | 0.29 - 0.66 | 0.25 - 0.56 | 0.22 - 0.59 | 0.10 - 1.11 |
|  | 0.38 ± 0.01 | 0.46 ± 0.01 | 0.49 ± 0.01 | 0.42 ± 0.07 | 0.44 ± 0.07 | 0.52 ± 0.23 |
| **Maximum diameter of the oral sucker, OSmax** | 0.53 - 1.06 | 0.63 - 1.14 | 0.79 - 0.98 | 0.57 - 1.03 | 0.60 - 0.99 | 0.51 - 1.17 |
|  | 0.76 ± 0.01 | 0.84 ± 0.01 | 0.88 ± 0.01 | 0.85 ± 0.07 | 0.83 ± 0.08 | 0.84 ± 0.14 |
| **Ventral sucker area, VSA** | 0.44 - 1.23 | 0.53 - 1.22 | 0.82 - 1.20 | 0.67 - 1.57 | 0.45 - 1.19 | 0.56 - 3.52 |
|  | 0.78 ± 0.01 | 0.89 ± 0.01 | 1.01 ± 0.02 | 1.01 ± 0.15 | 0.97 ± 0.10 | 1.98 ± 0.67 |
| **Maximum diameter of the ventral sucker, VSmax** | 0.75 - 1.25 | 0.89 - 1.29 | 1.07 - 1.35 | 0.92 - 1.49 | 0.69 - 1.24 | 0.87 - 1.91 |
|  | 1.00 ± 0.01 | 1.12 ± 0.01 | 1.18 ± 0.01 | 1.14 ± 0.10 | 1.13 ± 0.08 | 1.52 ± 0.19 |
| **OSA/VSA ratio** | 0.31 - 0.71 | 0.23 - 0.76 | 0.28 - 0.64 | 0.24 - 0.60 | 0.27 - 0.99 | 0.10 - 0.40 |
|  | 0.49 ± 0.01 | 0.52 ± 0.01 | 0.49 ± 0.01 | 0.42 ± 0.07 | 0.46 ± 0.09 | 0.25 ± 0.07 |
| **Distance between the anterior end of the body and the ventral sucker, A-VS** | 1.52 - 3.35 | 1.61 - 3.26 | 2.19 - 3.22 | 1.12 - 2.92 | 2.01 - 3.04 | 1.46 - 3.07 |
|  | 2.24 ± 0.02 | 2.48 ± 0.02 | 2.82 ± 0.03 | 2.09 ± 0.35 | 2.59 ± 0.19 | 2.38 ± 0.28 |
| **Distance between the ventral sucker and the posterior end of the body, VS-P** | 7.11 - 27.39 | 11.39 - 28.37 | 18.78 - 30.47 | 10.62 - 21.61 | 14.04 - 28.10 | 28.44 - 52.69 |
|  | 15.07 ± 0.31 | 16.47 ± 0.30 | 25.96 ± 0.43 | 16.61 ± 2.06 | 19.26 ± 3.63 | 39.13 ± 5.38 |

Lineal biometric characters in mm, areas in mm^2^ and ratios without units. **a** According to Bargues *et* al., 2021 **b** According to Periago *et al*., 2006.
