# Supplementary material for: Molecular characterization of Fasciola hepatica in endemic regions of Colombia
Source: Front Vet Sci. 2023 Jun 9;10:1171147. doi: 10.3389/fvets.2023.1171147 (PMC10288157; doi:10.3389/fvets.2023.1171147)
Supplement: Supplementary file 4 [file Table_4.DOCX]

***S4 Table.*** *Sequences and names of the primers used in the molecular characterization of F. hepatica in Antioquia, Boyacá, Cauca, Cundinamarca, Nariño, Norte de Santander, and Santander (Colombia).*

| **Marker** | **Primer** | **Sequences** | **References** |
| --- | --- | --- | --- |
| **28S** | FAS-28sFwd | AGCTGATTACCCGCTGAACT | Marcilla *et* al., 2002; Aghayan et al., 2019 |
|  | FAS-28sRv | CTGAGAAAGTGCACTGACAAG |  |
| **β-tubulina 3** | FAS-BtubFwd | CCCGGACAATTTTGTTTTCGGTCA | Teofanova *et al*., 2010 |
|  | FAS-BtubRv | CGTTGGTTCGGAATCCACTCGACAAA |  |
| **ITS1** | FAS-ITS1Fwd | TTGCGCTGATTACGTCCCTG | Shafiei *et al*., 2014 |
|  | FAS-ITS1Rv | TTGGCTGCGCTCTTCATCGAC |  |
| **ITS2** | FAS-ITS2Fwd | TGTGTCGATGAAGAGCGCAG |  |
|  | FAS-ITS2RV | TGGTTAGTTTCTTTTCCTCCGC |  |
| **COI** | FAS-COIFwd | ACGTTGGATCATAAGCGTGT | Itagaki *et al*., 2005; Schwantes et al., 2019 |
|  | FAS-COIRv | CCTCATCCAACATAACCTCT |  |
