# Supplementary material for: Molecular characterization of Fasciola hepatica in endemic regions of Colombia
Source: Front Vet Sci. 2023 Jun 9;10:1171147. doi: 10.3389/fvets.2023.1171147 (PMC10288157; doi:10.3389/fvets.2023.1171147)
Supplement: Supplementary file 5 [file Table_5.DOCX]

***S5 Table.*** *Accession Numbers of S. turkestanicum sequences used for phylogenetic and haplotype analyses*

| **Country** | **Acc. N°** | **Marker** | **Reference** |
| --- | --- | --- | --- |
| **Hungary** | JX467189 | *ITS1* | Lawton and Majoros (2013) |
| **Iraq** | LC214753 | *28s* | Suzan *et al.* (2017) |
| **Iran** | HM803240 | *ITS2* | Sadjjadi *et al*. (2012) |
| **UK** | OX103974.1 | *β-tubulina 3* | Berger (2022) |
| **Iran** | KC456232 | *COI* | Tabaripour and Youssefi (2013) |
