# Supplementary material for: Molecular characterization of Fasciola hepatica in endemic regions of Colombia
Source: Front Vet Sci. 2023 Jun 9;10:1171147. doi: 10.3389/fvets.2023.1171147 (PMC10288157; doi:10.3389/fvets.2023.1171147)
Supplement: Supplementary file 6 [file Table_6.DOCX]

***S6 Table.*** *Estimates of genetic divergence for COI and β-tubulin 3, number of sequences, and size of the fragments used. Colombian and external sequences from GenBank were included.*

| **Marker** | **Statistic** | | | | **Fragment size (bp)** | **Number of sequences** |
| --- | --- | --- | --- | --- | --- | --- |
|  | **h** | **hd** | **π** | **S** |  |  |
| ***COI*** | 5 | 0,684 | 0,00433 | 7 | 510 | 23 |
| ***β tub 3*** | 15 | 0,971 | 0,01323 | 31 | 818 | 19 |

| h: number of haplotypes, hd: haplotype diversity, π: nucleotide diversity and S: number of segregating sites. |
| --- |
