# Supplementary figures and images for: Molecular characterization of Fasciola hepatica in endemic regions of Colombia
Source: Front Vet Sci. 2023 Jun 9;10:1171147. doi: 10.3389/fvets.2023.1171147 (PMC10288157; doi:10.3389/fvets.2023.1171147)

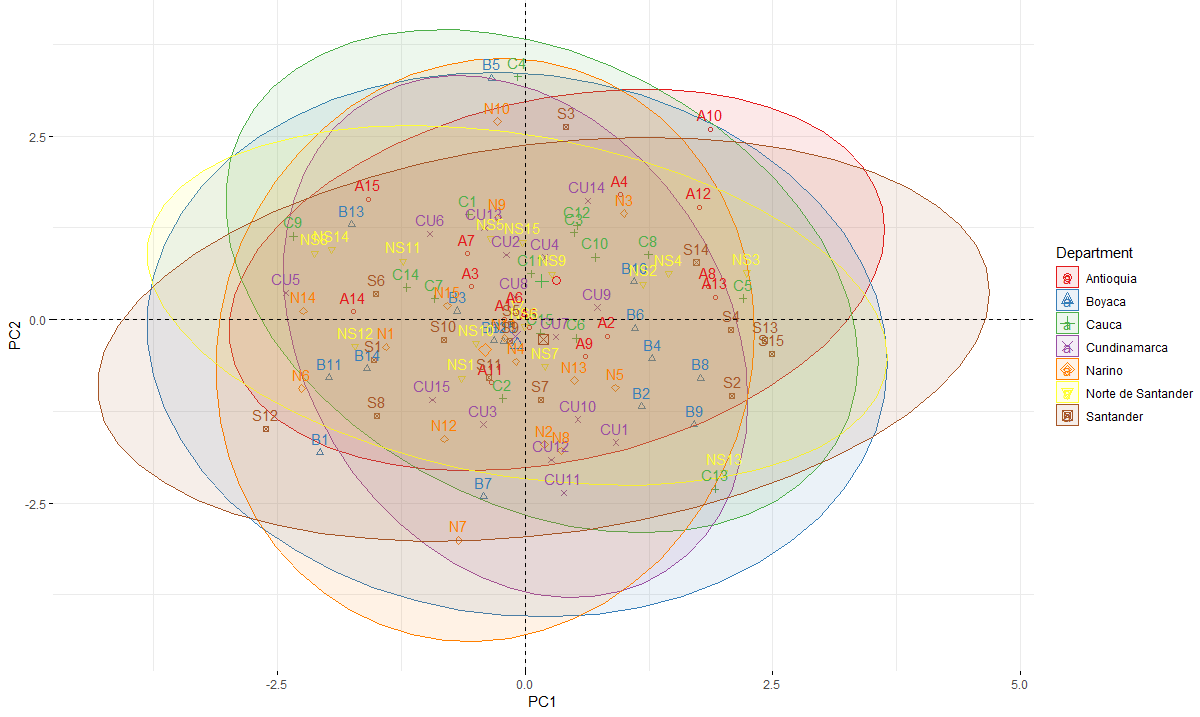

Supplement: Supplementary file 7 [file Image_1.PNG]

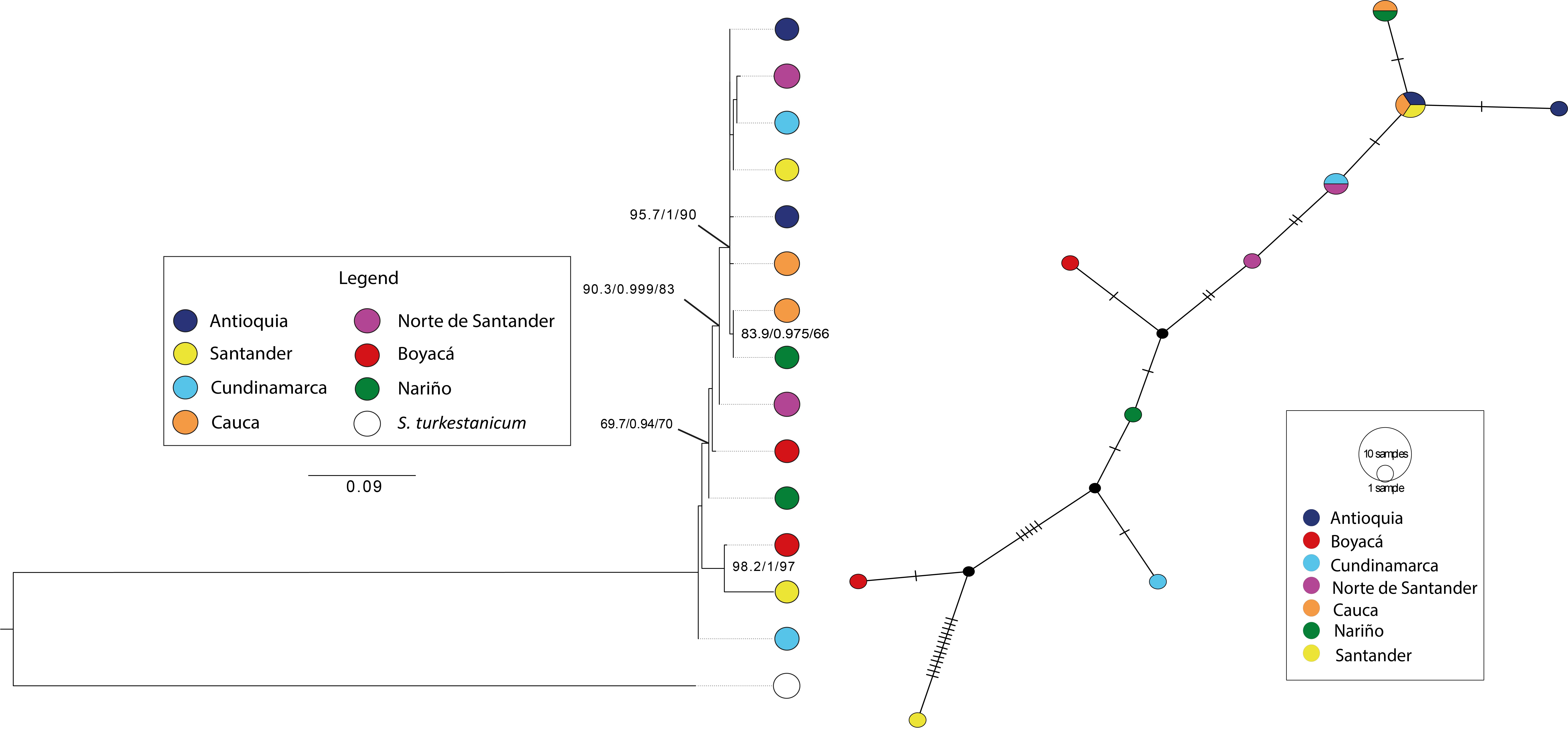

Supplement: Supplementary file 9 [file Image_3.JPEG]

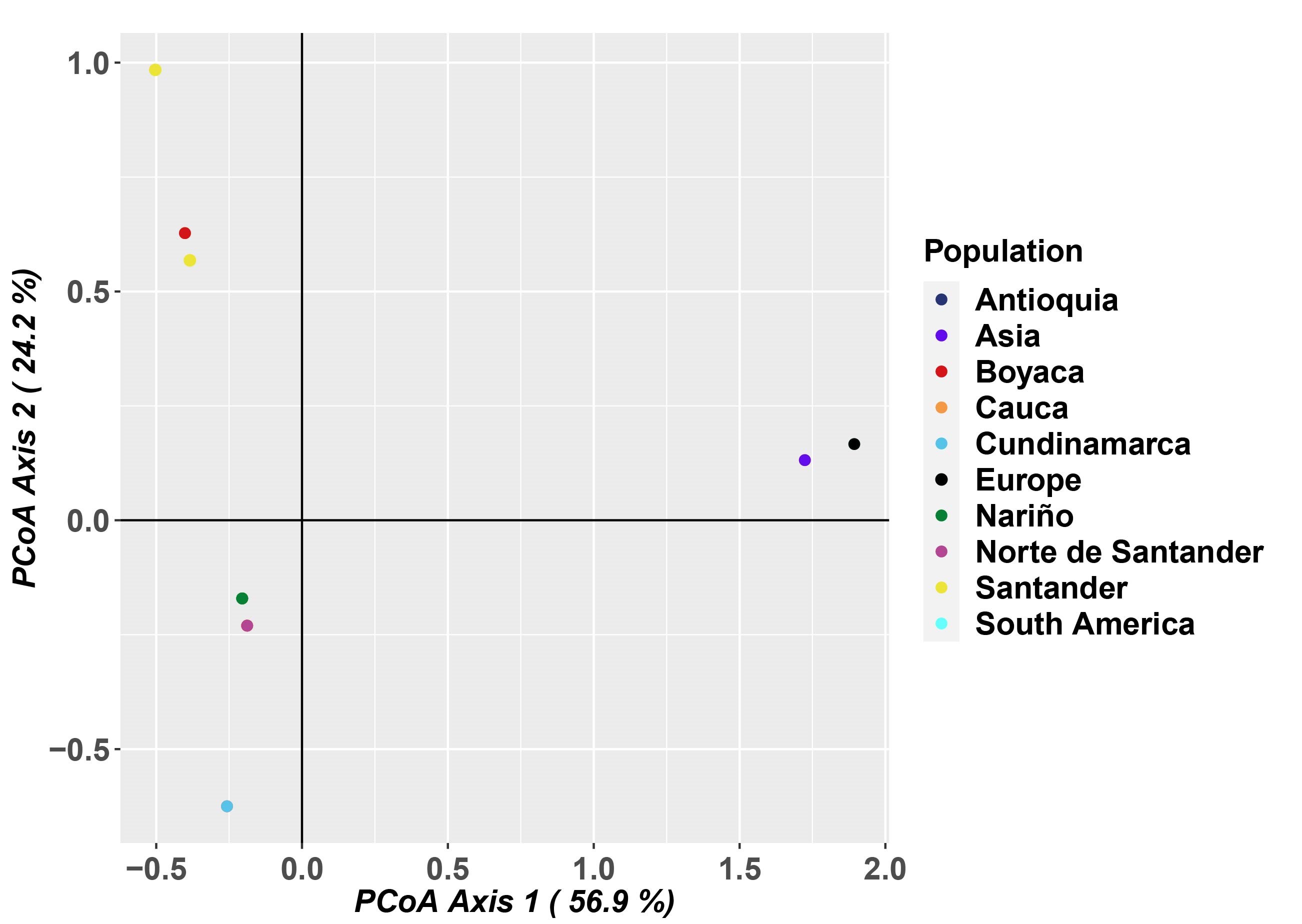

Supplement: Supplementary file 10 [file Image_4.JPEG]

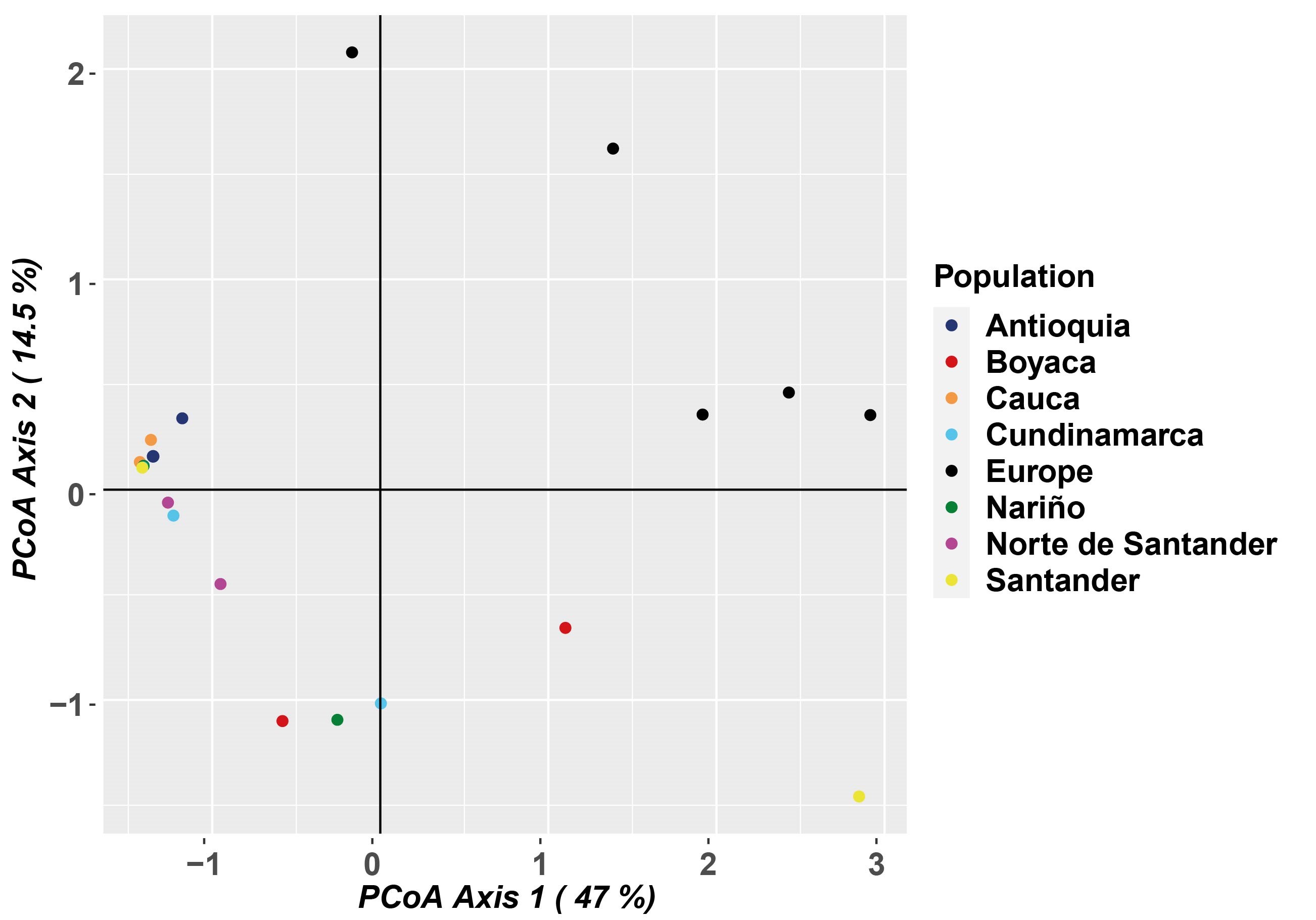

Supplement: Supplementary file 11 [file Image_5.JPEG]
